# Intrinsic and extrinsic feedback generate similar propulsion but distinct biomechanical strategies during split-belt walking

**DOI:** 10.3389/fnhum.2025.1729051

**Published:** 2026-01-06

**Authors:** Hamad K. Bin Shuwayyi, William R. Reed, Chen Lin, Jonathan D. Hill, Ann M. Varghese, Ya-Yu Liang, Christopher P. Hurt

**Affiliations:** 1 Rehabilitation Sciences Program, University of Alabama at Birmingham, Birmingham, AL, United States; 2 Department of Physical Therapy, Prince Mohammed Bin Abdulaziz Hospital, Riyadh Second Health Cluster, Riyadh, Saudi Arabia; 3 Department of Physical Therapy, University of Alabama at Birmingham, Birmingham, AL, United States; 4 Department of Neurology, Louisiana State University Health Shreveport, Shreveport, LA, United States; 5 Department of Biomedical Engineering, University of Alabama at Birmingham, Birmingham, AL, United States

**Keywords:** walking, kinetics, split-belt treadmill, joint power, feedback, trailing limb angle

## Abstract

**Background:**

Forward propulsion during walking is generated by different joints and biomechanical mechanisms depending on environmental and task demands. Although propulsion can be modulated by feedback, it is unclear whether extrinsic and intrinsic feedback generate similar propulsion or promote different joint-level strategies during split-belt walking. The purpose of this study was to investigate strategies used by non-impaired individuals to generate greater propulsion using different feedback to reach targeted levels of propulsion force during split-belt walking.

**Methods:**

Fifteen young adults walked on a split-belt treadmill with the dominant leg on the fast belt at their comfortable walking speed and the non-dominant leg on the slow belt at half speed. They performed trials with extrinsic via visual feedback of propulsive force (targeting 5 and 10% body weight) and with intrinsic feedback via a backward resistive force at the center of mass (5 and 10% body weight). Primary outcome was propulsion accuracy, measured as average propulsion error relative to target levels. Secondary analyses examined explanatory biomechanical variables related to propulsion generation. Outcomes were analyzed using two-way repeated measures ANOVA with Bonferroni correction.

**Results:**

Participants achieved similar target propulsion across feedback types (*p* = 0.66). However, biomechanical strategies differed. Visual feedback increased trailing limb angle (TLA) at 5% (*p* = 0.0011) and 10% (*p* < 0.0001) and increased ankle moment at 5% (*p* = 0.0005) and 10% (*p* < 0.0001). In contrast, backward resistive force increased (BRF) hip moment at 5% (*p* = 0.0018) and 10% (*p* < 0.0001), and hip power at both 5 and 10% (*p* < 0.0001). Ankle power did not differ between feedback types at 5% (*p* = 0.0754) but was greater under BRF at 10% (*p* < 0.0001).

**Conclusion:**

While both feedback types generate similar propulsion to achieve different target levels during split-belt treadmill walking, they engaged distinct biomechanical strategies. Our results indicate that participants increased TLA and ankle moment under visual feedback. However, they increased hip moment and hip power under BRF, with ankle power adjustments depending on the target level. The findings highlight motor abundance in gait and suggest tailoring rehabilitation strategies in populations with impaired propulsion.

## 1 Introduction

Walking is a complex activity requiring coordinated muscle actions of the limb to support and move the body forward step by step (Lacquaniti et al., 2012). Forward progression while walking is achieved primarily through the generation of positive mechanical work at the ankle and hip joint (Farris and Sawicki, 2012; Awad et al., 2020; Kuhman and Hurt, 2019a). For younger adults over level ground the generation of ankle joint moments and power generation are primary to forward progression (Winter, 1983; Neptune et al., 2001). In contrast to older adults, who show greater power production at the hip (DeVita and Hortobagyi, 2000; Franz, 2016). However, during tasks with greater external demand, such as uphill walking or stair ascent, the hip disproportionately increases its contribution to forward propulsion across participant groups by generating additional proximal power (Harper et al., 2018; Alexander et al., 2017). Kinematically, an increased trailing limb angle (TLA), which is the angle between the vertical axis and the line from the hip joint to the foot, also enhances forward propulsion (Lewek and Sawicki, 2019). Previous studies have demonstrated a strong correlation between the TLA and peak anterior ground reaction force (AGRF) (Hsiao et al., 2016; Lewek and Sawicki, 2019). Together, these joint mechanics, which are independently controlled, generate the horizontal ground reaction forces for forward progression of the center of mass (COM). The importance of the AGRF represents the force component that contributes most directly to forward movement (Nilsson and Thorstensson, 1989). We have previously shown that across walk speeds, timing of peak AGRF appears tightly controlled by the nervous system, but it is adaptable (Kuhman and Hurt, 2019b). The capacity of the nervous system of young adults to adapt (Seidler, 2006) by flexibly shifting joint work across limbs and joints to meet external demands (Hurt et al., 2022) makes it ideal to study propulsion without the confounds of aging or neurological injury.

While walking in the community, environmental constraints may force individuals to alter propulsion to maintain walking speed. As an example, walking up or down hills or walking on uneven ground. These constraints may require changes in propulsion that use different biomechanical strategies. Assessing the adaptability of propulsion and the strategies utilized may be important to understand mechanisms of action in modulating propulsion. One of the ways to modify propulsion bilaterally is by using split-belt treadmill walking, which involves two separate belts that can be set to different speeds. This experimental design provides a chance to evaluate how humans adapt propulsion in the event that each leg experiences different mechanical demands (Reisman et al., 2013; Chodkowska et al., 2024). Individuals can adapt on split-belt treadmills by extending the stance phase and reducing the swing phase on the slower leg. The increased contact time during split-belt walk provides additional opportunity to generate forward propulsion, especially during stance phase when ankle and hip joints contribute to force production (Reisman et al., 2005; Roemmich and Bastian, 2015). Recent studies involving split-belt treadmills have demonstrated immediate adaptations in timing and coordination both within and between legs. Because the limb on the slower belt has more time available for force generation, it becomes the ideal target for examining how individuals modify kinetic and kinematic output to meet external task demands, such as increasing propulsive force in response to feedback. Therefore, this study concentrated on the slower limb to isolate and analyze how biomechanical strategies differ across various feedback conditions.

Other methodologies exist to alter propulsion. Real-time feedback can deliver information about physiological processes that users may or may not consciously perceive, depending on the structure of that feedback (Stanton et al., 2017). Intrinsic feedback refers to the proprioceptive and somatosensory information generated internally from muscle spindles, Golgi tendon organs, joint receptors, and cutaneous afferents. These afferents continuously convey information about limb position, loading, and movement dynamics, supporting mostly automatic, reflexive adjustments during walking (Proske and Gandevia, 2012; Dietz, 2002; Dietz et al., 2002). During walking mechanical perturbations, such as backward resistive forces applied to the pelvis or limb, enhance proprioceptive load information and evoke compensatory adjustments to maintain locomotor tasks (Lewek et al., 2018; Park et al., 2020). In contrast, extrinsic feedback consists of externally provided cues, most commonly visual information, that explicitly inform individuals about their performance relative to a goal and facilitate conscious, voluntary motor corrections (Sigrist et al., 2013). During walking, extrinsic feedback via visual feedback is commonly used to guide modulation of propulsive forces or kinematic targets through real-time displays of anterior ground reaction force or limb trajectory (Hinton et al., 2024). Despite interest in using feedback to improve propulsion, it is still unclear whether different feedback types produce similar levels of propulsion generation. It also remains uncertain how distinct biomechanical strategies emerge under each modality during split-belt walking.

The purpose of this study was to investigate strategies used by nonimpaired individuals to generate greater propulsion using different types of feedback (i.e., intrinsic and extrinsic) to reach targeted levels of peak propulsion force for the limb on the slow belt during split-belt treadmill walking, where the two belts move independently at different speeds. It is important to investigate this important question first in healthy individuals to gain a basis of knowledge, which subsequently can be applied for targeted intervention in walking-impaired populations, such as post-stroke individuals. This can be best understood using the principle of motor abundance, which posits that the nervous system can achieve the same functional outcome through multiple biomechanical pathways (Latash, 2012). Based on this framework, we hypothesized that participants would achieve similar peak AGRF across feedback conditions (intrinsic vs. extrinsic) and target levels, but through different biomechanical strategies depending on the type of feedback and target level received. To test this hypothesis, healthy adults participated in walking trials under various feedback conditions on a split-belt treadmill.

## 2 Materials and methods

### 2.1 Participants

Fifteen young non-impaired adults (Table 1) consented to participate in this institutionally-approved study. Participants were free of neurological, cardiovascular, or musculoskeletal disorders, and able to walk independently. Individuals with any lower extremities’ injury within 6 months before the participation were excluded.

**Table 1:** Demographic and clinical characteristics of participants.

Variable	Value
Age (years)	25.47 ± 7.48
Height (cm)	172.01 ± 9.69
Weight (kg)	77.95 ± 21.60
CWS overground (m/s)	1.30 ± 0.13
CWS treadmill (m/s)	1.13 ± 0.20
Sex [Male]	11 (73%)
Dominant leg [R]	13 (87%)

The values mean ± SD for continuous variables and *n* (%) for categorical variables. The remaining sex and dominant leg proportions can be inferred. CWS, comfortable walking speed.

### 2.2 Instruments

**Figure 1:**
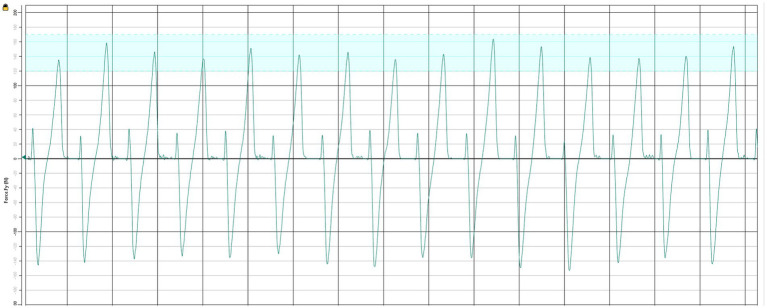
Setup for extrinsic feedback via the real-time visual interface. The visual feedback display presented step-by-step AGRF from the non-dominant limb. A horizontal target line with a shaded tolerance band indicated the participant-specific propulsion target (5% or 10% body weight).

**Figure 2:**
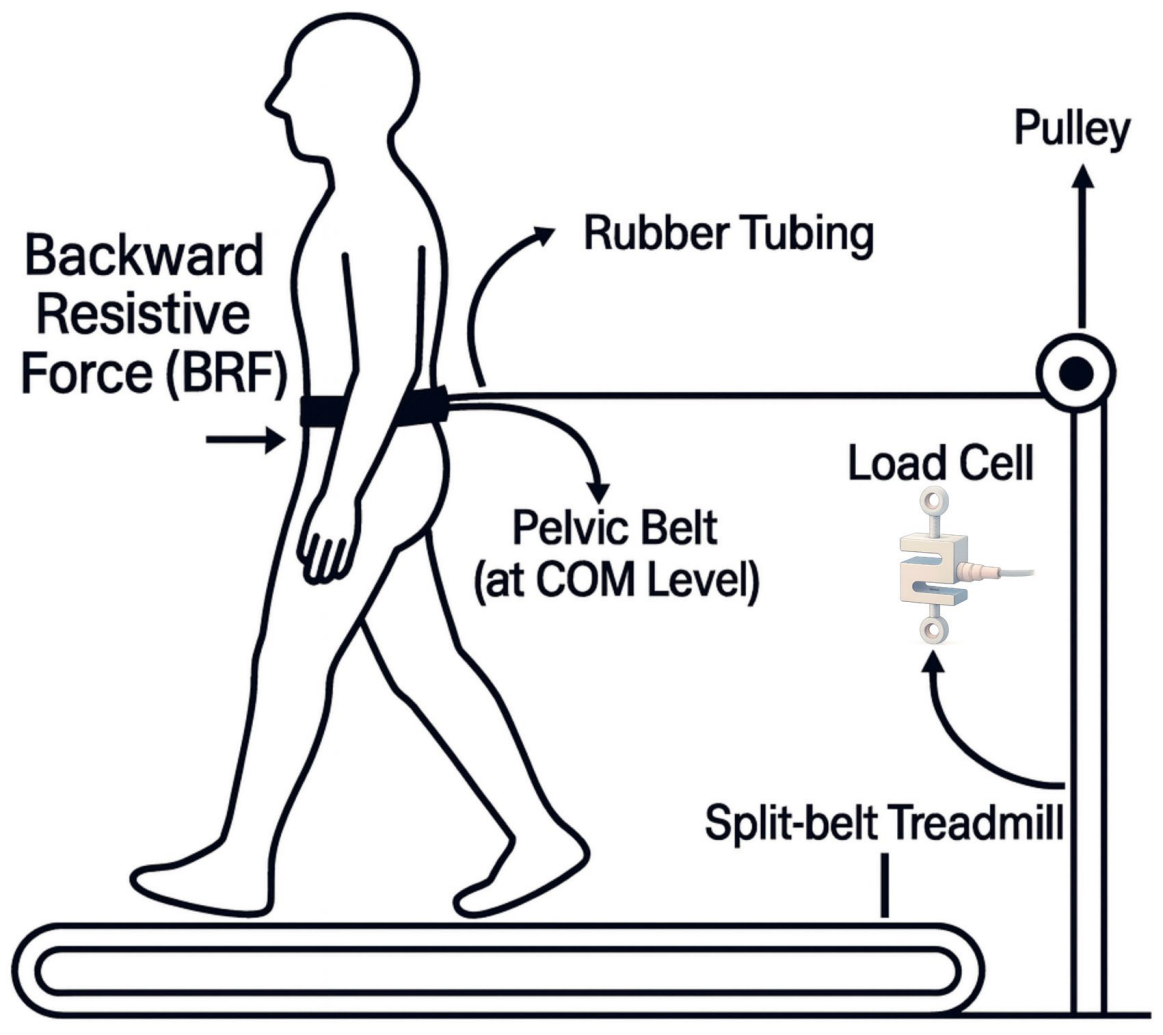
Setup for the intrinsic feedback condition using backward resistive force (BRF). A backward-directed resistive force was applied at the pelvis via a belt positioned at the level of the center of mass. The force was transmitted through rubber tubing routed around a pulley and monitored with an inline load cell.

We used a dual-belt, force-instrumented treadmill equipped with side hand railings (Motek, Amsterdam, BG). A ten-camera motion capture system (Vicon, Oxford EN) tracked passive reflective markers, that we processed offline (Vicon Nexus) to create a 12-segment rigid body biomechanical model. Real-time visual feedback was generated in Vicon Nexus using the built-in Monitor tool, which displayed the GRFs on a step-by-step basis and streamed the output to a graphical display. The feedback was delivered on a white screen positioned approximately 1.5 meters in front of the participant at eye level. The interface consisted of a vertical line that updated each step to represent the GRFs of the non-dominant limb, along with a horizontal target line overlaid with a shaded region indicating the participant-specific AGRF target (5% or 10% body weight). Participants were instructed to modulate their force output so that the bar reached the target line at each step (Figure 1). To create the backward directed resistive forces, we utilized a custom-built passive device that applied the resistive force through a belt won at the level of the center of mass (COM) (Figure 2). The custom device used rubber tubing fed through a winch to change force levels, which was measured with a load cell (RAS1-250S-S*C02, Loadstar Sensors Inc., Fremont, CA, USA) in series with the tubing. For safety, participants wore a fall harness that provided no support during treadmill walking but protected them if they lost their balance during the protocol.

### 2.3 Experimental protocol

Participant demographics, including weight, and dominant leg, were documented at the beginning of the session. Limb dominance was determined by participant self-report. Participants were asked which leg they naturally prefer for strength or balance tasks, like kicking a ball. Comfortable Walking Speed (CWS) was first measured overground on a Zeno gait mat (5.5 m × 0.91 m) and analyzed using ProtoKinetics Movement Analysis Software (ProtoKinetics, Havertown, PA). Participants completed two straight line walking passes across the walkway at their CWS. The instruction provided was: “Walk at a speed that feels most comfortable to you.” To ensure steady-state gait within the capture area on the mat, participants began walking 2 meters before the mat and continued 2 meters beyond it. After completing the first pass, they stopped, reset, and repeated the procedure for the second pass using the same instructions. Subsequently, each participant’s CWS on the treadmill was determined through a standardized protocol. Initially, the treadmill speed was set approximately 0.3 m/s slower than the participant’s overground speed and gradually increased in increments of 0.1 m/s until the participant reported, “This feels like my normal walking speed.” This speed was recorded, and the procedure was repeated starting at a speed faster than the previously identified speed, gradually decreasing by increments of 0.1 m/s until the participant again indicated their normal walking speed. The average of these two treadmill speeds was calculated and defined as the participant’s CWS for the treadmill condition (Dal et al., 2010; Martin et al., 1992).

Next, participants walked on the treadmill at their CWS for 1 min to obtain baseline measurements. Target Anterior Ground Reaction Forces (AGRFs) for visual feedback were then determined. Participants walked on the treadmill at half their CWS for 30 s, and the data was exported from Vicon Nexus software. This data was then analyzed using a custom script in MATLAB (MathWorks Inc., Natick, MA) to calculate the mean peak AGRFs for the non-dominant leg. The 5% visual feedback target AGRF was determined by adding 5% of the participant’s body weight to the mean peak AGRF of the non-dominant leg. The same procedure was followed to establish the 10% visual feedback target AGRF.


$$\text{Target}\;\mathrm{AGR}F_{\text{For visual feedback}}=\text{Mean Peak}\;\mathrm{AGR}F_{\text{Non}-\text{dominant}\;\mathrm{leg}}+\%\mathrm{BW}$$

After establishing the target AGRFs, participants completed an adaptation trial on the split-belt treadmill. The non-dominant leg was placed on the slow belt (set at half of the participant’s CWS), and the dominant leg was placed on the fast belt (set at the participant’s CWS). The adaptation trial lasted between 3 and 6 min. Initially, all participants completed a standardized 3-min adaptation period. After that, participants were asked whether they felt stable and comfortable, and the research team visually assessed gait for signs of instability or a lack of gait smoothness. For a few participants who reported continued discomfort or demonstrated observable instability, the adaptation time was extended in one-minute increments. Reassessment occurred after each minute, with the adaptation period ending once a stable walking pattern was observed. No participant required more than 6 min. This individualized approach ensured adequate acclimation to the split-belt condition before experimental testing.

Following this adaptation trial, participants completed an experimental protocol consisting of 6 trials, grouped into two feedback conditions (Intrinsic via backward resistive force and extrinsic via visual feedback). The order of feedback conditions was randomized to minimize adaptation and maintain engagement while target levels were delivered in a fixed ascending order within each feedback type. In addition, rest periods were provided between trials.

Before data collection, participants completed a familiarization with the visual interface while walking on the treadmill. During this orientation, they were shown how the GRF’s line height changed with their push-off force and limb position, and they practiced reaching the target line to ensure they understood the task. Participants were instructed to position their non-dominant leg behind their hip (increasing the trailing limb angle) and to push harder on the treadmill using their plantar flexors without holding the handrail. A similar familiarization was provided before the backward resistive force (BRF) condition, during which participants experienced the applied resistive load and were instructed on how to maintain their position at the center of the treadmill without using the handrails but use their legs to overcome the forces.

Intrinsic feedback: participants walked on the split-belt treadmill with the dominant leg on the fast belt at CWS and the non-dominant leg on the slow belt at half CWS, with backward resistive forces applied to the COM at 0, 5, and 10% of body weight (BW), respectively (Figure 2).

Extrinsic feedback: participants walked on the split-belt treadmill with the dominant leg on the fast belt at CWS and the non-dominant leg on the slow belt at half CWS while receiving real-time AGRF visual feedback at targets of 0% (no target), 5, and 10% of BW, respectively (Figure 1).

### 2.4 Data acquisition

Kinematic data was collected at 100 Hz using the capture motion system, while kinetic (ground reaction forces) were collected at 1000 Hz via the treadmill’s force plates. Both ground reaction forces and segment kinematic data were filtered in Vicon Nexus software (version 2.15, Vicon Motion Systems, Oxford, UK) using a 6 Hz and 12 Hz cutoff frequency for kinematics and kinetics, respectively. The filtered data were then exported for post-processing to Visual 3D (C-motion, Germantown, MD) and MATLAB (MathWorks Inc., Natick, MA).

For non-dominant limb (on slow belt), the peak AGRF was identified for every step as the maximum value of the anterior-directed force component (Fy) during the stance phase, defined from heel strike to toe-off with a threshold of 10 N. The mean peak AGRF was then calculated for the limb by averaging the peak propulsion values across all steps within each trial. After computing the peak anterior ground reaction force (AGRF) for each trial, we calculated the difference from the target AGRF by subtracting the mean peak AGRF of the leg on the slow belt from the predefined target value. This difference represented the participant’s error in achieving the target propulsion during each feedback condition.


$$\text{Difference from Target}=\text{Mean Peak}\;\mathrm{AGR}F_{\mathrm{leg}\;\mathrm{on}\;\text{slow belt}}-\text{Target AGRF}$$

Trailing limb angle was calculated as the angle between the vertical axis and the vector connecting the greater trochanter and lateral malleolus (ankle marker) in the sagittal plane at toe-off (Miyazaki et al., 2019). Toe-off was determined based on when the vertical ground reaction force dropped below 10 N, indicating the end of the stance phase and the start of the swing phase.

An inverse dynamic routine was used to quantify sagittal plane joint moments and positive powers at the hip and ankle, utilizing commercial software (Visual 3D, C-Motion Inc., Germantown, MD). For each step, the moments and positive power were calculated during the stance phase (from heel strike to toe-off) and normalized to the individual’s body mass.

## 3 Statistical analyses

All statistical analyses were performed using RStudio (version 2024.12.0 + 467). To test our hypothesis, a two-way repeated measures ANOVA was used to assess the study’s primary outcome variable, average propulsion error, comparing two feedback types across two target levels. A significance level of *p* < 0.05 was used for this analysis. Since only a single test was conducted for this primary measure, no adjustment for multiple comparisons was applied.

Secondary analyses were conducted on explanatory variables related to our primary outcome. We performed five separate two-way repeated measures ANOVAs to examine the effects of feedback type (intrinsic vs. extrinsic), target level (0, 5, and 10% body weight), and their interaction on the following dependent variables: trailing limb angle (TLA), ankle moment, hip moment, ankle power, and hip power. To account for multiple comparisons across these five explanatory outcomes, a Bonferroni correction was applied, resulting in a significance level of *p* < 0.01. We used this method to reduce the risk of Type I error since we are assessing multiple related tests. When assumption of sphericity was violated by Mauchly’s test, the Greenhouse–Geisser correction was used. For any significant main effects or interactions, *post hoc* pairwise comparisons were performed, and adjusted *p*-values are listed.

## 4 Results

### 4.1 Average propulsion error (difference from target)

**Figure 3:**
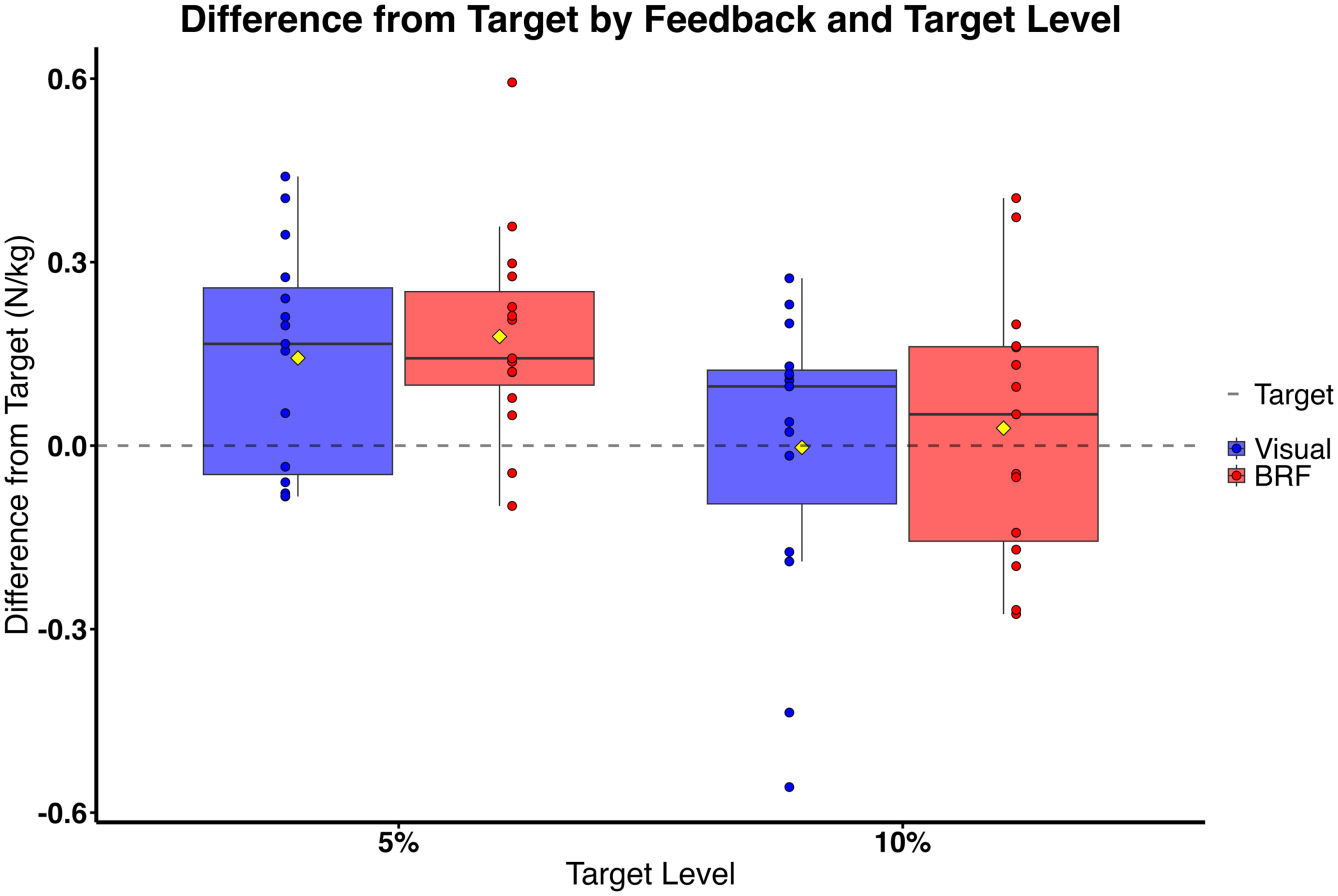
Box plots show the difference from the target AGRF (N/Kg) for limb on slow belt during split belt walking across visual feedback (blue) and backward resistive force (BRF: red) at 5% and 10% body weight target level. The dashed horizontal line represents target matching. Yellow diamond represents the mean. Individual data points overlayed. No significant main effect of feedback (*p* = 0.66) or feedback × target interaction (*p* = 0.94) was observed.

Both intrinsic and extrinsic feedback conditions yielded similar propulsive force outputs. The average error from the target was summarized as mean ± SD at each of the two target levels. There was no significant main effect of feedback on average propulsion error (*F* (1, 14) = 0.20, *p* = 0.66) with a small effect size (ηp^2^ = 0.014), indicating that participants achieved similar accuracy in reaching the target propulsion level under both visual and backward resistive force (BRF) conditions. At the 5% target level, the mean error was 0.14 ± 0.18 N/kg for visual feedback and 0.18 ± 0.17 N/kg for BRF. At the 10% target level, the mean was 0.00 ± 0.24 N/kg for visual feedback and 0.03 ± 0.22 N/kg for BRF. These findings suggest that participants were able to match the target propulsion level similarly under both feedback conditions (Figure 3).

### 4.2 Trailing limb angle (TLA)

**Figure 4:**
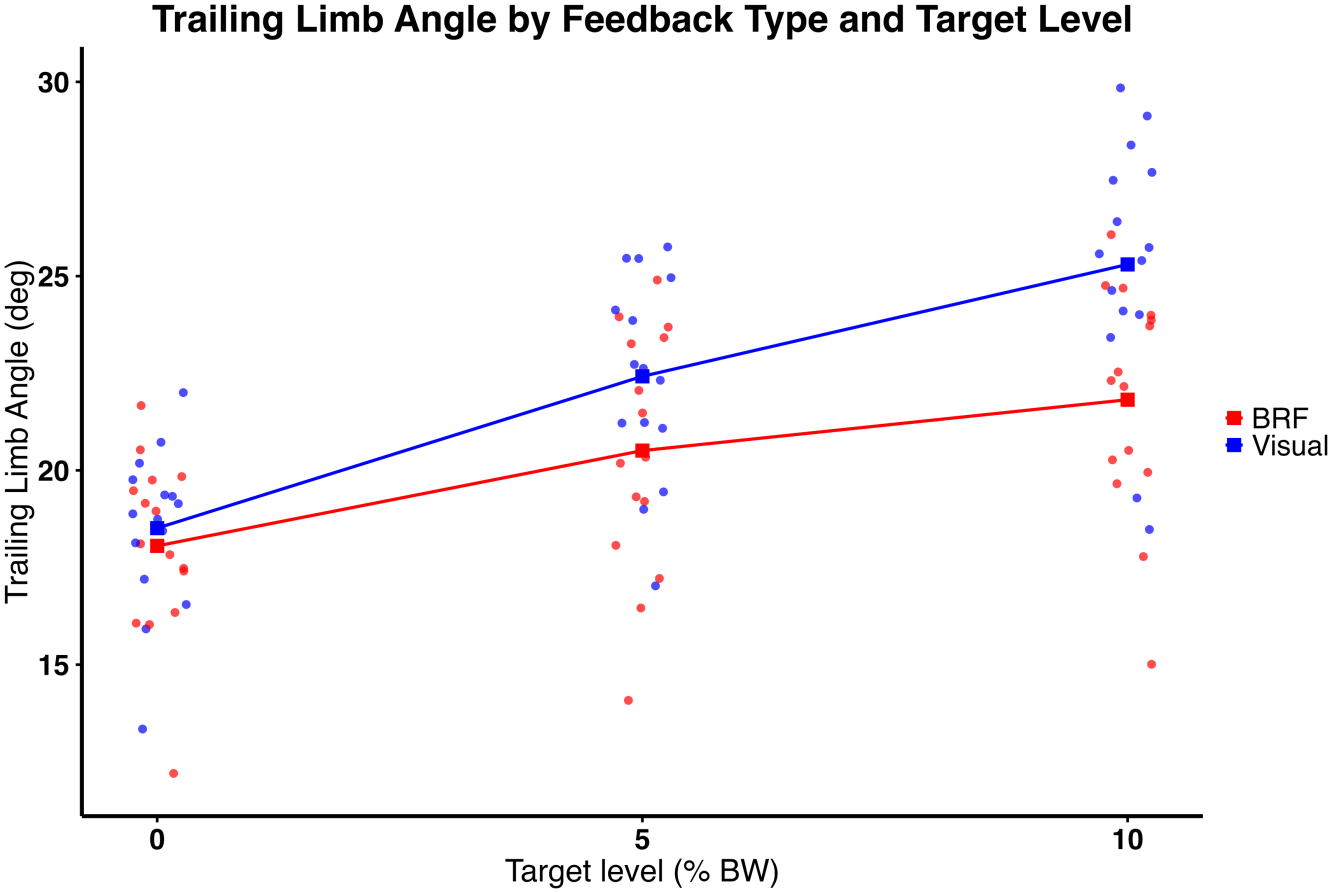
Trailing limb angle (°) for the limb on the slow belt during split-belt treadmill walking under visual feedback (blue) and backward resistive force (BRF, red) across 0%, 5%, and 10% body weight target levels. Individual data points overlayed (dots), with squares and connecting lines representing group means. Significant feedback × target interaction was observed (*p* = 0.0002), indicating that the effect of target level on TLA depended on feedback type. Visual feedback produced greater TLA than BRF (*p* = 0.0003), and TLA increased significantly across target levels (*p* < 0.0001).

**Figure 5:**
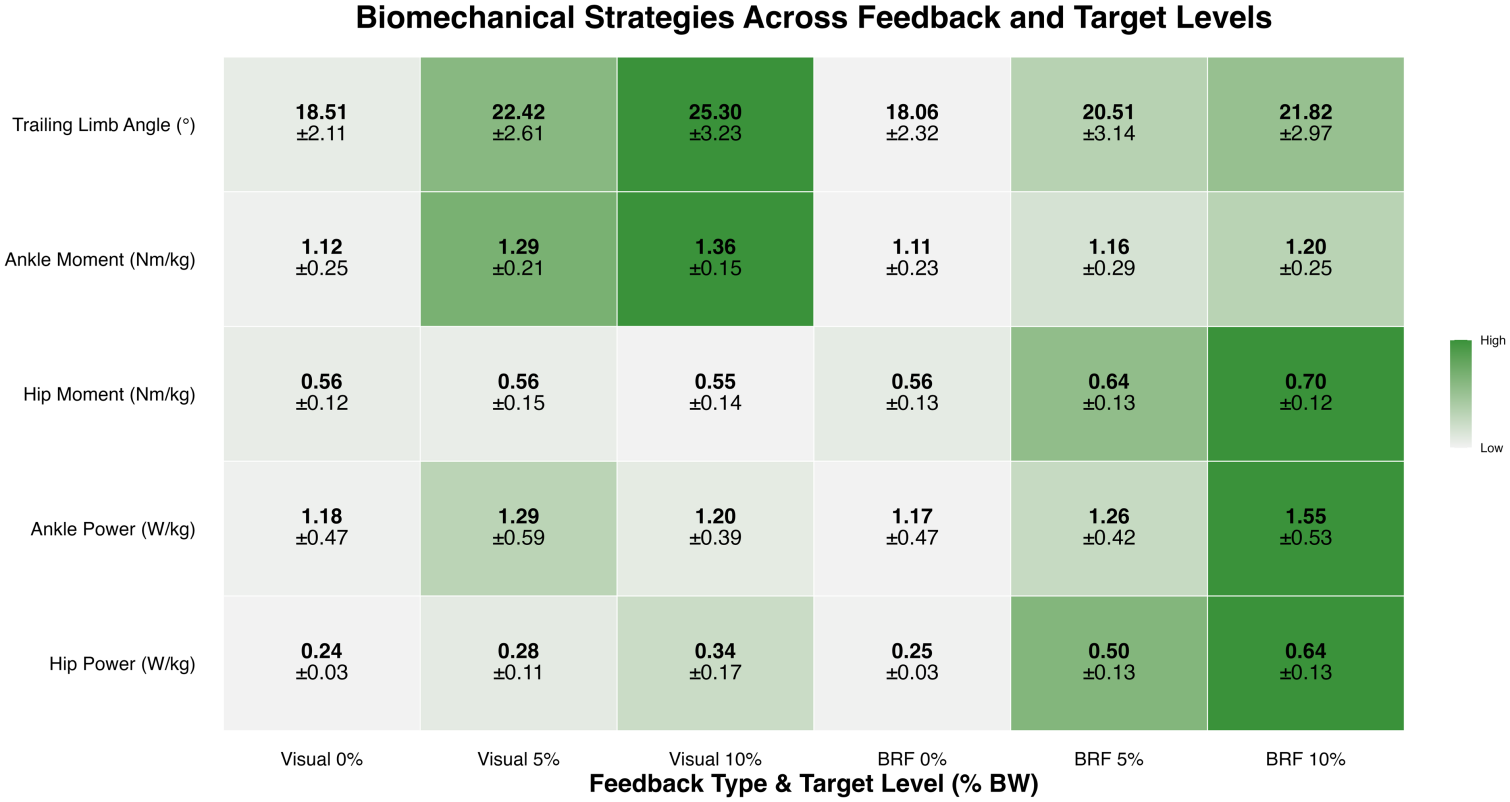
Mean ± SD values for trailing limb angle (°), ankle moment (Nm/kg), hip moment (Nm/kg), ankle power (W/kg), and hip power (W/kg) for the limb on the slow belt during split-belt treadmill walking under visual feedback and backward resistive force (BRF) across 0%, 5%, and 10% body weight target levels. Color intensity represents the magnitude of each variable, with darker shades indicating higher values. Statistical analyses revealed significant main effects of feedback and target level for all variables except ankle power, which showed no main effect of feedback but a significant feedback × target interaction.

Trailing limb angle was moderated by type of feedback and amount of force. As shown in Figure 4, we found significant feedback × target interaction (*F* (2, 28) = 15.76, *p* = 0.0002), indicating that the effect of target level on TLA depended on feedback type. *Post hoc* comparisons with Bonferroni correction revealed that, at each target level, visual feedback produced significantly greater TLA than BRF. At the 5% target, TLA was 22.42 ± 2.61° for visual and 20.51 ± 3.14° for BRF (t (80, 35) = −3.39, *p* = 0.0011) with a medium effect size (η^2^ = 0.12). At the 10% target, TLA was 25.30 ± 3.23° for visual and 21.82 ± 2.97° for BRF (t (80, 35) = −6.18, *p* < 0.0001) with a large effect size (η^2^ = 0.21). In addition, a significant main effect of target level was observed (F (2, 28) = 72.88, *p* < 0.0001), with TLA increasing incrementally across the 0, 5, and 10% targets. A main effect of feedback was also present (*F* (1, 14) = 22.04, *p* = 0.0003), with visual feedback producing greater TLA overall compared to BRF. Descriptive results (mean ± standard deviation) are presented in Figure 5.

### 4.3 Ankle moment

**Figure 6:**
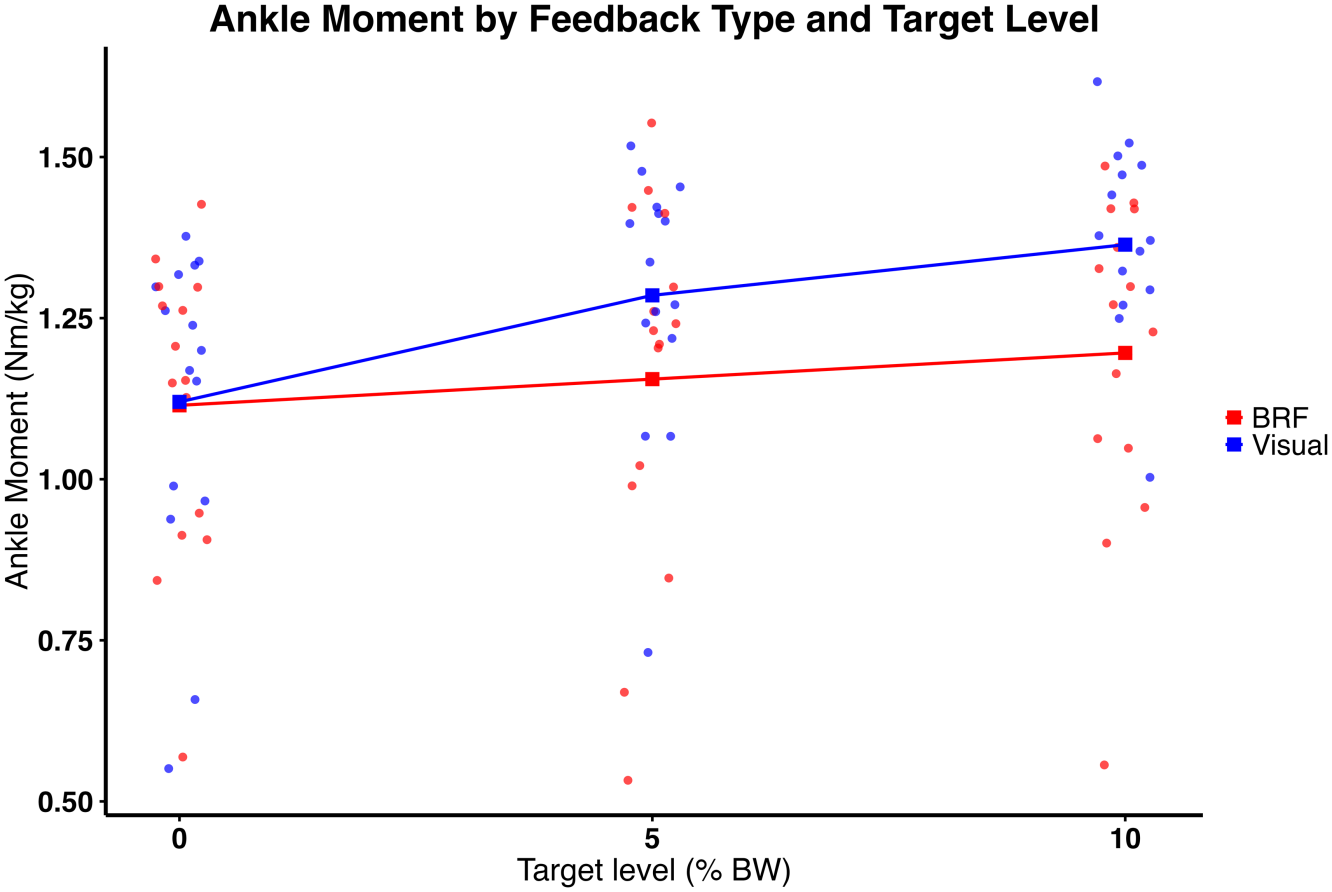
Ankle moment (Nm/kg) for the limb on the slow belt during split-belt treadmill walking under visual feedback (blue) and backward resistive force (BRF, red) across 0%, 5%, and 10% body weight target levels. Individual data points overlayed (dots), with squares and connecting lines representing group means. A significant feedback × target interaction was observed (*p* = 0.0087). A significant main effect of feedback was also present, with visual feedback producing greater ankle moments overall compared to BRF (*p* = 0.0011). In addition, a significant main effect of target level was observed, with ankle moment increasing incrementally at higher targets (*p* < 0.0001).

Ankle moment was moderated by type of feedback and amount of force. As shown in Figure 6, significant feedback × target interaction was observed (*F* (2, 28) = 5.65, *p* = 0.0087), indicating that the effect of target level on ankle moment differed depending on feedback type. In addition, a significant main effect of target level was observed (*F* (2, 28) = 27.19, *p* < 0.0001), with ankle moment increasing incrementally as propulsion targets increased. A main effect of feedback was also present (*F* (1, 14) = 16.64, p = 0.0011), with visual feedback producing greater ankle moments overall compared to BRF. *Post hoc* comparisons with Bonferroni correction revealed that visual feedback elicited significantly greater ankle moment than BRF at both the 5 and 10% target levels. At the 5% target, the mean ankle moment was 1.29 ± 0.21 Nm/kg for visual feedback and 1.16 ± 0.29 Nm/kg for BRF (t (80, 35) = −3.63, *p* = 0.0005) with a medium to large effect size (η^2^ = 0.14). At the 10% target, ankle moment was 1.36 ± 0.15 Nm/kg for visual feedback and 1.20 ± 0.25 Nm/kg for BRF (t (80, 35) = −4.69, *p* < 0.0001) with a large effect size (η^2^ = 0.21). These results suggest that participants used more ankle joint moments under visual feedback. Descriptive results (mean ± standard deviation) are presented in Figure 5.

### 4.4 Hip moment

**Figure 7:**
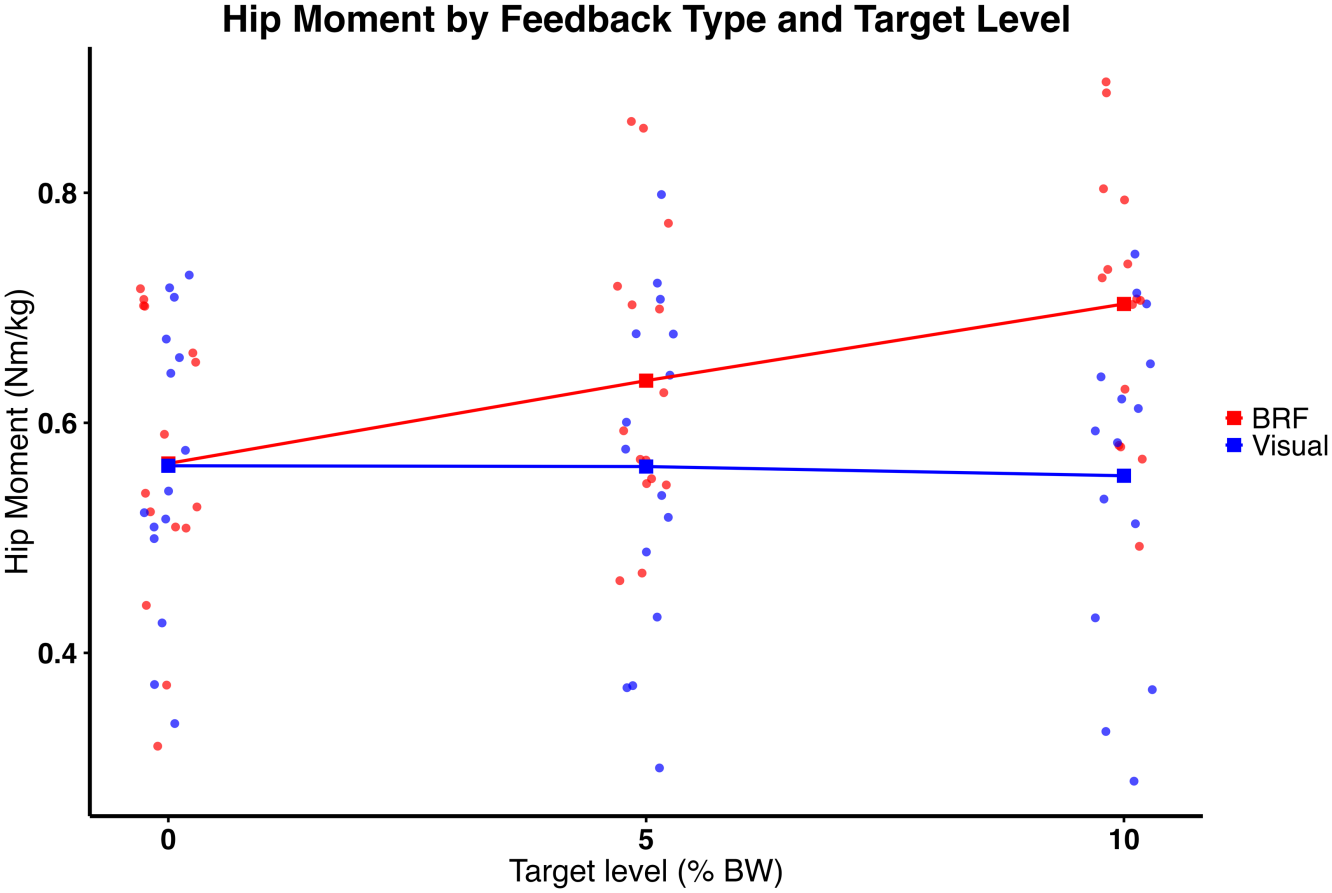
Hip moment (Nm/kg) for the limb on the slow belt during split-belt treadmill walking under visual feedback (blue) and backward resistive force (BRF, red) across 0%, 5%, and 10% body weight target levels. Individual data points overlayed (dots), with squares and connecting lines representing group means. Significant feedback × target interaction was observed (*p* = 0.0004), indicating that the effect of target level on hip moment differed between feedback types. There were significant main effects of feedback (*p* = 0.0039) and target level (*p* < 0.0001).

Hip moment was moderated by type of feedback and amount of force. As shown in Figure 7, a significant feedback × target interaction was observed (*F* (2, 28) = 14.76, *p* = 0.0004), indicating that the way hip moment changed with target level depended on feedback type. In addition, a significant main effect of target level was observed (*F* (2, 28) = 17.22, *p* < 0.0001), with hip moment increasing as propulsion targets increased. A main effect of feedback was also present (*F* (1, 14) = 11.88, *p* = 0.0039), with BRF producing greater hip moments overall compared to visual feedback. *Post hoc* comparisons with Bonferroni correction revealed that BRF elicited significantly greater hip moment than visual feedback at both the 5 and 10% target levels. At the 5% target, the hip moment was 0.64 ± 0.13 Nm/kg for BRF and 0.56 ± 0.15 Nm/kg for visual feedback (t (80, 35) = 3.23, *p* = 0.0018) with a medium effect size (η^2^ = 0.11). At the 10% target, hip moment increased to 0.70 ± 0.12 Nm/kg under BRF and 0.55 ± 0.14 Nm/kg under visual feedback (t (80, 35) = 6.47, *p* < 0.0001) with a large effect size (η^2^ = 0.34). Descriptive results (mean ± standard deviation) are presented in Figure 5.

### 4.5 Ankle power

**Figure 8:**
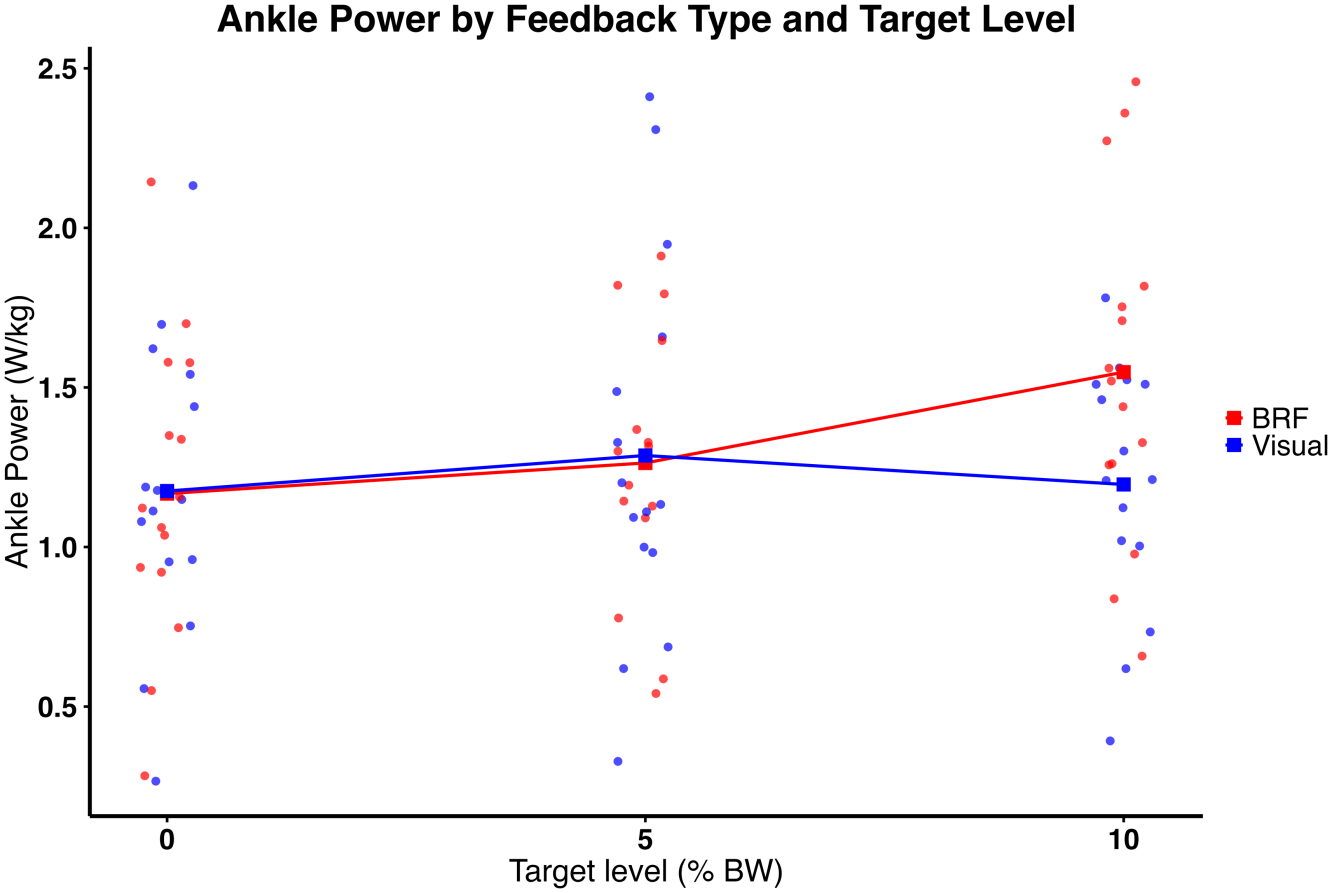
Ankle power (W/kg) for the limb on the slow belt during split-belt treadmill walking under visual feedback (blue) and backward resistive force (BRF, red) across 0%, 5%, and 10% body weight target levels. Individual data points overlayed (dots), with squares and connecting lines representing group means. Significant feedback × target interaction was observed (*p* = 0.0019), indicating that the effect of feedback on ankle power varied depending on the target level. A significant main effect of target level was also present (*p* = 0.0050), with ankle power increasing as propulsion targets increased. There was no significant main effect of feedback (*p* = 0.0389).

Ankle power was moderated by type of feedback and amount of force. As shown in Figure 8, significant feedback × target interaction was present (*F* (2, 28) = 7.94, *p* = 0.0019), indicating that the effect of feedback on ankle power varied depending on the propulsion target level. In addition, a significant main effect of target level was observed (F (2, 28) = 8.34, *p* = 0.0050), with ankle power increasing as propulsion targets increased. There was no significant main effect of feedback (*F* (1, 14) = 5.19, *p* = 0.0389). *Post hoc* comparisons with Bonferroni correction revealed that, at 5%, there was no significant between feedback types (t (80, 35) = −0.31, *p* = 0.0754). The average ankle power was 1.29 ± 0.59 W/kg under visual feedback and 1.26 ± 0.42 under BRF. However, at 10%, the average ankle power was 1.20 ± 0.39 W/kg under visual feedback and 1.55 ± 0.53 under BRF. Ankle power was significantly greater under BRF compared to visual feedback (t (80, 35) = 4.73, *p* < 0.0001) with a large effect size (η^2^ = 0.21). These results suggest that BRF enhances ankle power generation specifically when the propulsion demand is high. Descriptive results (mean ± standard deviation) are presented in Figure 5.

### 4.6 Hip power

**Figure 9:**
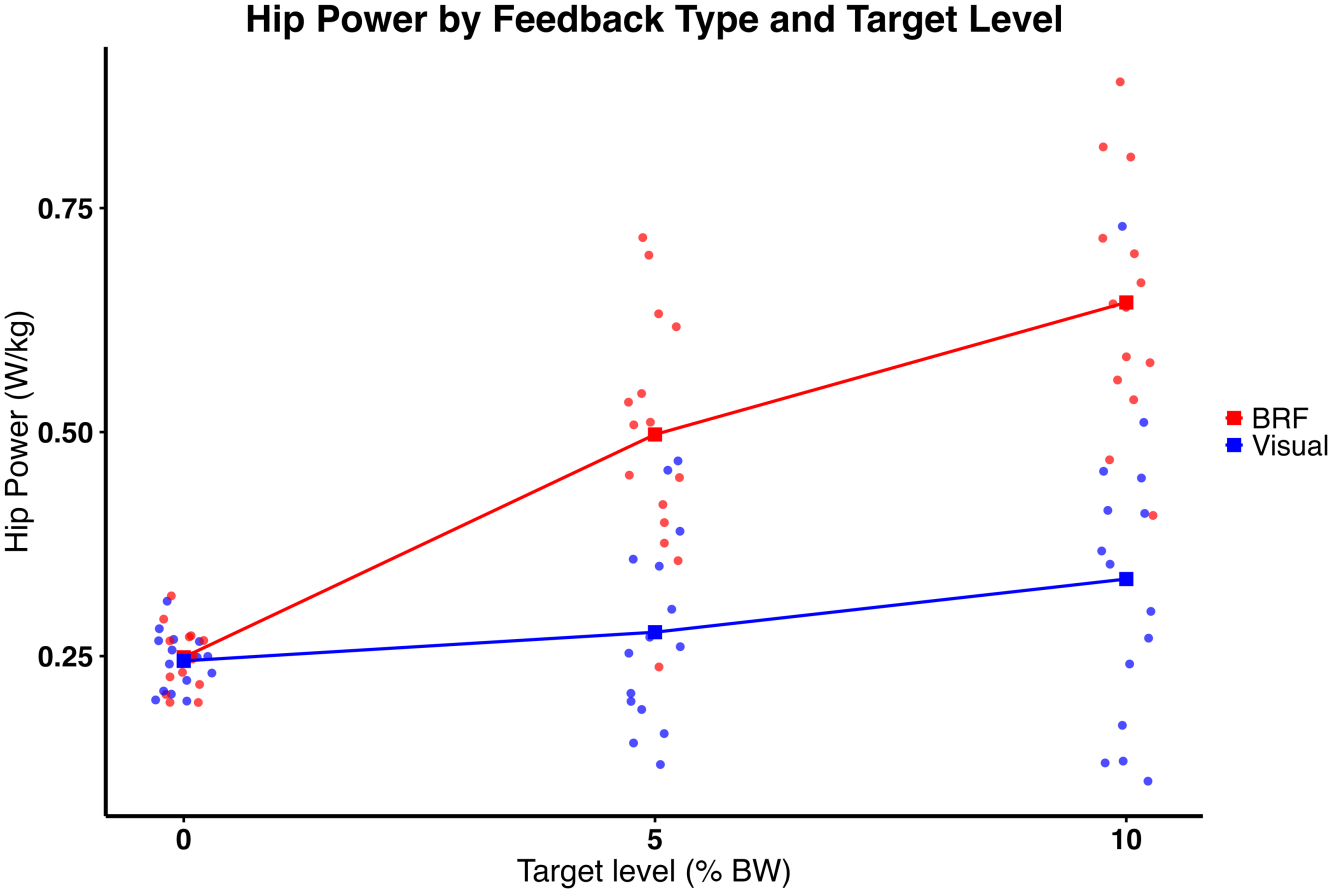
Hip power (W/kg) for the limb on the slow belt during split-belt treadmill walking under visual feedback (blue) and backward resistive force (BRF, red) across 0%, 5%, and 10% body weight target levels. Individual data points overlayed (dots), with squares and connecting lines representing group means. A significant feedback × target interaction was observed (*p* < 0.0001), indicating that the effect of feedback on hip power varied across target levels. Significant main effects of feedback (*p* < 0.0001) and target level (*p* < 0.0001) were observed, with hip power being greater under BRF and increasing with higher propulsion targets.

Hip power was moderated by type of feedback and amount of force. As shown in Figure 9, significant feedback × target interaction was present (F (2, 28) = 27.11, p < 0.0001), indicating that the effect of feedback on hip power varied depending on the propulsion target level. In addition, a significant main effect of target level was observed (F (2, 28) = 61.51, *p* < 0.0001), with hip power increasing incrementally as propulsion targets increased. A main effect of feedback was also present (F (1, 14) = 45.43, p < 0.0001), with BRF producing greater hip power overall compared to visual feedback. Post hoc comparisons with Bonferroni correction revealed that, at the 5% target, hip power was 0.50 ± 0.13 W/kg under BRF and 0.28 ± 0.11 W/kg under visual feedback. This difference was statistically significant (t (80, 35) = 6.45, *p* < 0.0001) with a large effect size (η^2^ = 0.34). At the 10% target, hip power further increased under proprioceptive feedback to 0.64 ± 0.13 W/kg, while it remained lower under visual feedback at 0.34 ± 0.17 W/kg. This difference was also significant (t (80, 35) = 9.02, *p* < 0.0001) with a large effect size (η^2^ = 0.50). Descriptive results (mean ± standard deviation) are presented in Figure 5.

## 5 Discussion

The purpose of our study was to investigate strategies used by nonimpaired individuals to generate greater propulsion using different feedback (i.e., intrinsic and extrinsic) to reach targeted levels of peak propulsion force on the slow belt during split-belt treadmill walking. We hypothesized that participants would achieve similar peak AGRF across feedback conditions using different biomechanical strategies depending on the type of feedback and target level received. In support of our hypothesis, participants were able to match the target propulsion level similarly under both feedback conditions, using different strategies to reach different target levels. For clarity, we refer to extrinsic feedback as visual feedback, and intrinsic feedback as backward resistive force (BRF). Under visual feedback, individuals increased the TLA and ankle moment, whereas under backward resistive force individuals increased hip moment and hip power, and at the higher propulsion target, peak ankle power production. To the best of our knowledge, this is the first study to compare biomechanical strategies under intrinsic and extrinsic feedback during split-belt walking, providing a foundational understanding for interventions targeting propulsion in individuals with gait impairments.

Our observation that both types of feedback generate similar propulsion is not unexpected. For example, Liu et al. (2020) failed to find significant difference between three types of feedback (audio, visual, audiovisual) in peak propulsive force generation while walking in both healthy and stroke population. This finding is consistent with previous research showing that individuals adjust their force output in response to increasing external demands during split-belt treadmill walking. For example, it has been shown that propulsive impulse is increased by adding backward-directed resistance, particularly in the slow leg of a split-belt condition, highlighting the adaptability of the neuromotor system to meet the demands of a task (Moradian et al., 2023). Moreover, our current study did reveal that real time visual feedback increased TLA as the target level increased, which is consistent with a study showing that visual kinematic feedback improves TLA (Hinton et al., 2024). In addition, participants in our study increased their ankle moment more under visual feedback compared to BRF. A similar finding was observed in the post-stroke population, where BRF did not elicit increases in peak ankle moment (Lewek et al., 2018). We observed that participants increased their ankle moment in BRF, but to a lesser degree than under visual feedback.

Increases in TLA and ankle moment observed under visual feedback as the target level increased more than BRF may reflect the role of visual–motor integration in guiding precise, goal-oriented adjustments to meet external targets or demands. Real-time visual feedback provides continuous information about propulsion generation relative to targeted force, allowing participants to consciously position the limb further behind the body at toe-off, thereby increasing TLA. This posterior positioning optimizes the orientation of the ground reaction force vector, enabling a greater ankle plantarflexor moment during late stance when pushing harder on the ground or treadmill (Lewek and Sawicki, 2019). Furthermore, during visual feedback familiarization, participants received explicit instructions to position their non-dominant leg behind the hip, a cue that was not given in the BRF condition. Given that earlier research has demonstrated that verbal cues can cause immediate and quantifiable changes in gait patterns (Parker et al., 2022), this instruction probably contributed to the higher TLA values seen under visual feedback. On the other hand, increases in hip moment and power under BRF compared to visual feedback may be due to the backward force application at the COM which challenges forward progression to maintain the participant’s position on the treadmill while maintaining walking speed. Proprioceptive feedback from muscle spindles and joint mechanoreceptors delivers continuous information about limb position, load, and body orientation (Proske and Gandevia, 2012; Neptune et al., 2001; Farris and Sawicki, 2012). Since the BRF is applied around the pelvis, it primarily engages postural control pathways that recruit proximal muscles, such as the hip extensors, rather than relying only on distal ankle control (Winter, 1995; Kuo, 2002). In addition, from a mechanical perspective, the application of force is closer to the hip joint center and more easily countered by increasing force output of the hip extensors thus creating a larger internal extensor hip moment. Resisting this perturbation biases participants under BRF toward producing greater hip extensor moment to stabilize the trunk and pelvis (Winter, 1995; Zajac, 1989) while also generating greater propulsion force to overcome the added force so that the individual can stay in the center of the treadmill. Therefore, we observed increased hip moment and positive power to generate forward propulsion and resist forward trunk flexion (maintaining trunk and pelvic stability). Moreover, ankle power differed significantly under BRF compared to visual feedback at the higher target level (10% BW) but not at the lower target level (5% BW). This pattern may represent a secondary mechanism to increase propulsion once hip extensor output reaches a functional limit, ensuring the additional force demand can be met without compromising stability (Neptune et al., 2001; Zajac et al., 2003).

Surprisingly, our study did not reveal a significant effect of feedback type on ankle power for the 5% condition, even though ankle moment and trailing limb angle (TLA) were clearly different for visual vs. BRF. One potential reason for this finding is that visual feedback may have increased the ankle moment, but it might not have increased the ankle’s angular velocity in late stance. If the moment is higher but angular velocity of the ankle does not increase, power production at the ankle may not increase with higher force targets (Farris and Sawicki, 2012). This suggests that under visual feedback, participants positioned the foot further behind the COM (larger TLA) directing the ground reaction force forward as they pushed harder against the treadmill. Moreover, the limb we focused on was on the slower belt, increasing stance time while individuals focused attention on matching a visual target. Thus, individuals did not have to optimize ankle angular velocity while walking on the treadmill. These factors together help explain why ankle power did not change, even though there were significant increases in ankle moment and TLA.

Our findings from this study have clinical implications for gait rehabilitation, specifically, populations with impaired propulsion such as post-stroke individuals. Gait rehabilitation can be an indicator of future functional outcomes and quality of life (Lin et al., 2021). The observation that participants achieved similar levels of peak anterior ground reaction force AGRF using different biomechanical strategies under different feedback conditions highlights the flexibility of the neuromotor system. This suggests that propulsion targets can be achieved by exploiting the lower limb multiple degrees of freedom that can provide a unique solution to a movement problem, supporting the principle of motor abundance and providing a basis for developing tailored rehabilitation strategies (Latash, 2012). Notably, the proximal emphasis observed under BRF is consistent with an established age-related shift in propulsion, in which older adults increasingly rely on the hip as ankle power declines (DeVita and Hortobagyi, 2000; Franz, 2016). Although the present study did not explicitly address aging, acknowledging this distal-to-proximal redistribution informs our understanding of how different feedback modalities may influence propulsion strategies. It shows how the locomotor system’s propulsion strategies can change in response to mechanical or sensory demands. For instance, extrinsic feedback via visual feedback may be used when the goal is to enhance trailing limb angle and ankle moment, which are often reduced in individuals with plantarflexor weakness or limited limb extension. On the other hand, intrinsic feedback via BRF may be used for promoting proximal contributions, such as hip moment and hip power, which are important for trunk stability and forward advancement in those with distal impairments. However, additional studies involving larger populations of individuals with impaired nervous systems are needed before recommending widespread clinical implementation.

### 5.1 Limitation

The current study has several limitations that should be discussed. First, we acknowledge that our estimation of CWS may not fully reflect each participant’s true overground preferred walking speed. However, because this study used a repeated-measures design in which all feedback conditions (intrinsic and extrinsic) were tested relative to each participant’s own treadmill-derived CWS, any potential inaccuracy would have influenced all conditions equally. Thus, we believe our primary outcomes remain comparable across conditions. Second, during familiarization with visual feedback, participants were instructed to place their non-dominant leg behind their hip to help them reach the AGRF target, but not in BRF, as reflected in TLA results. Regardless, these differences likely contributed to the distinct biomechanical strategies observed but did not affect the primary outcome. Participants achieved similar propulsion accuracy across conditions. Third, no neural-level measurements, such as EMG, cortical and subcortical imaging, etc., were collected in this study. Although our biomechanical variables capture the primary mechanical contributors to propulsion, they do not allow direct inference about the underlying neural control processes. Nevertheless, since our interpretations focus on mechanical strategy selection rather than neural control mechanisms, the lack of neural-level measurements does not affect our primary conclusions. In addition, our analysis was limited to propulsion strategies quantified through TLA and ankle and hip joint kinetics. Although these variables represent the primary mechanical pathways for generating forward propulsion, they do not capture knee joint contributions, segmental mechanical work, or muscle activation patterns. However, the group-level results clearly showed different biomechanical strategies across feedback conditions while participants achieved similar levels of propulsion, even without measuring all possible propulsion contributors. Participants walked under the split-belt paradigm for only 3 to 6 min in the adaptation trials, which may not have allowed complete adaptation of the gait pattern to reach a steady state. Previous studies have shown that locomotor adaptation, especially in step length symmetry, can continue for a long time in a novel split-belt paradigm (Sánchez et al., 2019). However, differences become relatively small after initial strides of adaptation (Reisman et al., 2007), and we have previously shown that kinetic measures can adapt quickly during short split-belt exposures (Hurt et al., 2022). Therefore, while prolonged walking may reveal additional adaptations, our short adaptation trials were sufficient to capture immediate joint-level strategies under feedback. The duration of gait trials with feedback was short (1 min) and differences between the types of feedback may potentially arise during longer-duration walking trials. However, our question focused on the immediate effect of feedback rather than long-term adaptation. Split-belt treadmill and feedback studies show that changes in different gait outcomes and propulsion measures can occur within minutes or even on the initial exposure (Vasudevan and Bastian, 2010; Reisman et al., 2010; Liu et al., 2020). Lastly, we limited assessing outcomes to the slow belt only of the split-belt walking trial. However, several studies have already shown how these types of feedback influence propulsion and joint-level strategies during tied-belt walking (Kuhman and Hurt, 2019a; Browne and Franz, 2018). Therefore, our conclusions were restricted to the split-belt walking paradigm by design.

### 5.2 Conclusion

This study provides insight into how individuals with a non-impaired nervous system can generate similar peak AGRF to achieve different target levels during split-belt treadmill walking under extrinsic (visual) and intrinsic (BRF) feedback yet rely on distinct biomechanical strategies. Our results indicate that participants increased TLA and ankle moment under visual feedback, whereas they increased hip moment and hip power under BRF, with ankle power adjustments depending on task demand or target level. It would be of interest to perform follow-up studies in larger samples of healthy adults and in post-stroke individuals. These studies should investigate and compare both feedback types during split-belt treadmill walking. Focus should be given to how the paretic limb employs different biomechanical strategies to achieve various propulsion targets.

## Data Availability

The raw data supporting the conclusions of this article will be made available by the authors, without undue reservation.
